# Temporal Response of Mesocarnivores to Human Activity and Infrastructure in Taihang Mountains, Central North China: Shifts in Activity Patterns and Their Overlap

**DOI:** 10.3390/ani13040688

**Published:** 2023-02-16

**Authors:** Yanzhi Chen, Beibei Liu, Deqing Fan, Sheng Li

**Affiliations:** 1 Keystone Academy, Beijing 101318, China; 2 Chinese Felid Conservation Alliance, Beijing 101121, China; 3 Tieqiaoshan Provincial Nature Reserve, Jinzhong 032704, China; 4 School of Life Sciences, Institute of Ecology, Peking University, Beijing 100871, China

**Keywords:** niche partitioning, camera-trapping, circadian rhythm, temporal overlap, human disturbance, temperate montane forest, carnivores

## Abstract

**Simple Summary:**

Humans alter how carnivores interact with one another by changing landscapes and inciting fear. We investigated how four mesocarnivores (medium-sized carnivores), the red fox, leopard cat, Asian badger, and hog badger, partition their activity pattern to co-occur under varying human influences in the Taihang Mountains of China. Using camera-trapping data collected from 2016 to 2020, we revealed that the leopard cats and the badgers reduced their activities during the day at sites with high-level human disturbance, possibly a behavioral mechanism to avoid risks while living in human-dominated landscapes. However, the activity pattern overlap did not increase between mesocarnivore pairs, suggesting that they may use strategies other than niche segregation along the temporal dimension to coexist.

**Abstract:**

Mesocarnivores play essential roles in terrestrial ecosystems, but anthropocentric disturbances have profoundly transformed their intraguild interactions worldwide. In this study, we explored how a guild of four mesocarnivores (red fox *Vulpes vulpes*, leopard cat *Prionailurus bengalensis*, Asian badger *Meles leucurus*, and hog badger *Arctonyx collaris*) partition their temporal niche in the temperate montane forests in North China under different human influences. We conducted a systemic camera-trapping survey on the study species in the central Taihang Mountains from 2016 to 2020. With an extensive survey effort of 111,063 camera-days from 187 camera stations, we obtained 10,035 independent detections of the four mesocarnivores and examined the activity patterns of each species under different levels of human disturbance and their overlaps. The results showed that, while the leopard cat and the badgers shifted their activity towards nocturnality, the red fox showed no significant change. The leopard cat’s degree of nocturnality varied between growing and non-growing seasons, likely a response to avoid humans and other competitors. However, the activity overlaps between species pairs demonstrated no statistically significant difference, indicating a long-developed coexistence mechanism that is homogenous across the landscape. Demonstrating how mesocarnivores shift activity patterns in response to human risks while partitioning resources, this study enhances our understanding of mesocarnivore behavioral changes and interspecific interactions at human–nature interfaces.

## 1. Introduction

Over the past century, humans have transformed most of Earth’s ecosystems and encroached on significant extents of the wilderness [1], driving global species loss [2], changing animal behaviors [3], and fundamentally influencing the ecological processes of terrestrial ecosystems [4,5]. To avoid risks associated with human “super-predation”, wild animals respond on spatio-temporal dimensions [6,7] to both the immediate presence of humans and human landscape modification such as roads and buildings [8], hitherto referenced as “activity” and “infrastructure”, respectively [9]. This behavioral change creates a “landscape of fear” [10] and refines wildlife’s realized niche space [11], which will further alter interspecific interactions [12] and influence the niche partitioning among co-existing animals.

The sympatry of carnivores can be facilitated through the differentiation of ecological niches along different dimensions [13,14] in space (e.g., [15]), time (e.g., [16]), and diet (e.g., [17]). In particular, plasticity in the temporal niche axis is integral to the segregation of competing carnivores, which may reduce their direct encounters and potential overlap on dietary resources [18,19], a common and easy behavioral strategy to relax the interspecific competition and therefore facilitate their coexistence [20].

In human-dominated landscapes, various human disturbances further complicate the niche partitioning within the community of multiple carnivore species [3], because human activities can directly or indirectly influence carnivore population densities, drive risk avoidance, and change resource distribution [16,21]. For instance, Gaynor et al. [10] found a global shift to nocturnality among wildlife as a result of human disturbance. Such changes in response could disrupt the equilibrium of coexistence and reconstruct wildlife communities [22]. Human disturbance could reduce niche space, thus increasing niche overlap and degrading niche partitioning, leading to more intense competition that may decrease species fitness [23,24]. In addition, different carnivore species exhibit differential tolerance to human impacts [25]. With less tolerant species facing a trade-off between energy-efficient foraging and avoidance of human impacts, tolerant species may benefit by expanding their dietary and habitat breadth [3].

However, despite various studies that investigated the individual behavioral responses of carnivores under human disturbance, few studies have examined how such a non-lethal response shapes interactions within animal guilds [7,26,27]. Specifically, how the time dimension of the ecological niche shapes community interactions is poorly understood in carnivore communities, and the seasonality of such responses also remains to be uncovered [8].

Mesocarnivores are small- to medium-sized species (<15 kg) in the Carnivora family [28] that play important ecological roles in terrestrial ecosystems. They can potentially influence vegetation structure, cause trophic cascades, and fill the niche spaces following the elimination of large carnivores, a common phenomenon in many degraded ecosystems [28,29]. However, relatively few studies have independently explored the dynamics of mesocarnivore sympatry and its potential implications [28,30,31]. With rapidly increasing human encroachment, this knowledge gap highlights growing research needs to inform species interaction and predict cascade effects on the trophic web [32].

In this study, we examined the interspecific temporal niche partitioning dynamics within a diverse mesocarnivore community living in temperate montane forests in North China. With similar activity patterns, size, and spatial occupancy [33,34,35,36], the studied sympatric mesocarnivores are expected to exhibit intra-guild competition and partitioning. We established a camera-trapping network across the study area to evaluate how the temporal patterns and levels of temporal niche partitioning between mesocarnivores are influenced by differential levels of human activity and infrastructure. We mainly tested three hypotheses: (1) wildlife would show increased nocturnal activities and decreased diurnal activities with high human disturbance; (2) temporal overlap among mesocarnivores increases with an increased human disturbance at both large- and fine-scale, and (3) the patterns of niche partitioning are subject to seasonal changes.

## 2. Materials and Methods

### 2.1. Study Area

**Figure 1:**
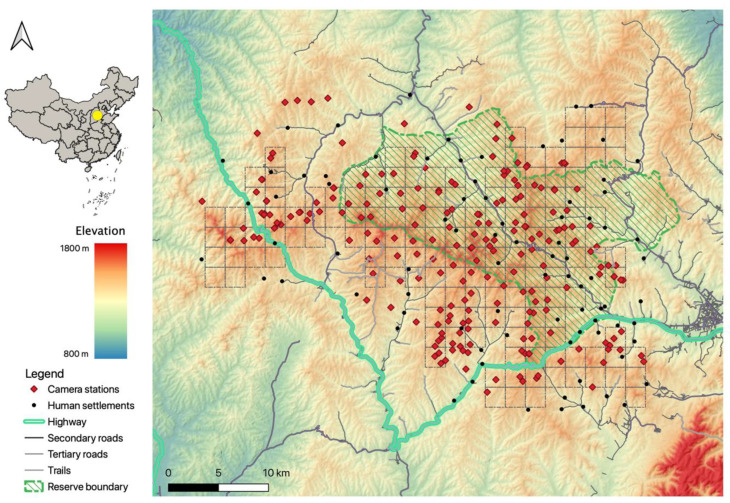
The study area and all camera survey stations during the period 2016–2020 at the Tieqiaoshan Nature Reserve, Shanxi Province, China.

The study was conducted at the Tieqiaoshan (TQS) Provincial Nature Reserve and the surrounding areas, in Shanxi Province, China. The TQS reserve is located in the central Taihang Mountains, at 113°04′–113°22′ E, 37°22′–37°34′ N with an area of 353.5 km^2^ (Figure 1). The annual precipitation is 700 mm [34] and the annual mean temperature is 7.3 °C. The monthly mean temperature in the hottest month, July, reaches 21.6 °C, while the coldest, January, has a monthly mean temperature of −9.1 °C [37]. The climate can be roughly divided into the growing season (from May to October) and the non-growing season (from November to April).

The reserve has a homogenous landscape, with an elevation ranging from 1300 m to 1800 m and a temperate montane forest ecosystem that is dominated by the Chinese red pine (*Pinus tabuliformis* Carriére), the North China larch (*Larix principis-rupprechtii* Farjon), white birch (*Betula platyphylla* Sukaczev), and the Liaotung oak (*Quercus liaotungensis* Koidz) [33,34]. Inside the reserve, there are 47 villages with a total of approximately 9000 inhabitants [38], who mainly utilize the reserve for farming, herb gathering, and logging [39]. Corn farming and cattle grazing are their primary livelihoods. The study area is scattered, with one national highway, two secondary roads (large paved roads), numerous tertiary roads (small paved roads), and unpaved roads.

Previous studies have recorded eight carnivore species in the TQS reserve, among which the North China leopard (*Panthera pardus japonensis*) is the apex predator [40,41]. The other seven are mesocarnivores, including two canines, the red fox (*Vulpes vulpes*) and raccoon dog (*Nyctereutes procyonoides*), one felid, the leopard cat (*Prionailurus bengalensis amurensis*), three mustelids, the Asian badger (*Meles leucurus*), hog badger (*Arctonyx collaris*), and the Siberian weasel (*Mustela sibirica*), and one viverrid, the masked palm civet (*Paguma larvata*) [40,41]. The raccoon dog, Siberian weasel, and masked palm civet are rare in this area and are therefore excluded from this study.

### 2.2. Data Collection

We adopted a grid design by dividing the study area into 152 2 km × 2 km sampling cells, and then established an array of camera-trapping survey stations to record animal activities (Figure 1). We set up one to four camera stations in each cell. To minimize spatial autocorrelation, camera stations were deployed at least 500 m from each other [23,40,42]. Each station was mounted with one motion-triggered camera trap (L710; YiAnWS-Loreda R&D Center, Shenzhen, China). To maximize detection, cameras were placed by animal or human trails without bait or lure [43], attached to trees or rocks at 30–90 cm above the ground, and set with a 30-s between consecutive triggers. Camera data were collected approximately once every two months. Each image (picture or video) was considered one record and was processed to extract the information of capture date, time, station ID, and species of the record. We grouped successive images of the same species captured by a camera within a 30-min interval as one independent detection to avoid pseudo-replication of the data [23,44,45], regardless of the number of individuals captured in that detection [23].

### 2.3. Human Disturbance Factors

Human disturbance factors are measured in three dimensions, including human activity, distance to roads, and distance to settlements. The intensity of human activity at each survey station was measured by the human’s relative abundance index (RAI), which was calculated as the photographic rate (i.e., the number of detections per day averaged over the total number of camera-days) of humans captured by the camera [46]. We also calculated the distance to roads and human settlements of each station, respectively, as two variables that could impact the animal’s behavior. Settlements are identified through high-resolution satellite imageries (Google Earth v. 7.3.2.5776., 2020), and roads are mapped with OpenStreetMap (https://planet.openstreetmap.org, accessed on 1 October 2021). Because both paved and unpaved roads are frequented by cars, we assumed that they exert similar ecological impacts and thus combined them as “roads” in our analysis. The distance to the nearest roads and settlements was calculated using QGIS (v. 3.0, QGIS Development Team) [47,48].

### 2.4. Data Analysis

We examined the activity patterns of the four mesocarnivore species with sufficient detection, namely the leopard cat, red fox, Asian badger, and hog badger. Due to the difficulty in distinguishing the two badger species from the camera footage in most cases, they were grouped as “badger” in subsequent analyses.

#### 2.4.1. Activity Overlaps under Large-Scale Human Disturbance

To address our first hypothesis, we compared the temporal responses at high and low human disturbance levels. To facilitate a more direct comparison of human disturbance across a gradient of human pressure, we used the *K*-means clustering method [49] to categorize human disturbance, in terms of activity, distance to road, and distance to settlement, into separate levels, following the suggestions of Sévêque et al. [8]. The algorithm divides *N* sites into *K* clusters so that the within-cluster sum of squares is minimized [49]. The data were first scaled with a standard deviation of 1 and a mean of 0. Following the method established by Kabacoff [50], we conducted six tests, namely Hubert’s Statistic, a “wssplot” function, D-index value, the Elbow method, the Silhouette method, and the Gap Statistic Method, from packages *factoextra* [51], *cluster* [52], and *NbClust* [53], in R with the data of each disturbance factor separately to determine the optimal number of clusters to be formed for each variable [54]. The final number of clusters was taken as the mode suggested by the six statistical tests. The same test was performed on the three human disturbance measures independently. Regardless of the number of clusters formed, the highest category (high disturbance) and lowest category (low disturbance) were used for analysis.

We modeled the activity pattern of each study species with a non-parametric kernel density function using the *overlap* package in R [55,56]. This model assumes that individual detections arise from a continuous probability density function [9]. We first converted time into radians, then generated the activity pattern function of each study species at each human disturbance level. We then quantified the overlap between activity curves of each species pair using the overlap coefficient, a value that ranges from 0 to 1 [56], which is calculated with the sample size appropriate method with 10,000 bootstraps to obtain a 95% confidence interval [56]. We first obtained the function and overlap coefficient on how the three mesocarnivores species groups offset at the two human disturbance levels among the three disturbance variables. To quantify the shift in mesocarnivore activity with human activity, the overlap coefficient between humans and each mesocarnivore was calculated with a 95% confidence interval obtained from bootstrapping. In addition, we modeled the function at the growing season (i.e., May to October) and non-growing season (i.e., November to April), respectively, to examine whether the activity overlap differed between seasons for specific species pairs.

We would expect a lower overlap coefficient if the activity pattern is influenced by human disturbance, and a higher value if not. Then, to assess the impact of changes in temporal patterns on niche partitioning, we calculated the overlap coefficient at each human disturbance level for each species pair. A higher value at high human disturbance would indicate the degradation of temporal niche partitioning. This would allow comparisons of how the strength of niche partitioning changes with different levels of human disturbance.

To determine statistical significance in the shift of the overlap coefficients between mesocarnivore pairs and humans at high and low human activity, we conducted a Welch’s T-test using the bootstrapped overlap coefficient distribution [57]. The sample size used in the t-test was consistent with the number of mesocarnivore detections at each human activity level.

#### 2.4.2. Activity Overlaps under Fine-Scale Human Disturbance

We conducted a mixed-effect regression analysis to examine how human disturbance characteristics at each site influence the temporal partitioning between mesocarnivore pairs using the package *nlme* [8,23,58]. We constructed the mixed-effect regression with road distance, village distance, and activity as fixed effects to determine how human disturbance shapes the temporal activity overlap coefficient (Δ) on a continuous scale. Village ID and road ID are used as the mixed effects because each village differs in its population, main livelihood, and level of proximate development, and each road differs in its width and traffic, influencing the level of human development. The model is shown below:

(1) $$\Delta =\beta_{0}+\beta_{1}\left(activity\right)+\beta_{2}\left(road\right)+\beta_{3}\left(village\right)+\gamma_{1}\left(village\;ID\right)+\gamma_{2}\left(road\;ID\right)+\varepsilon_{i}$$

The best model was determined in a top-down fashion by comparing the likelihood ratio *L* calculated using ANOVA [59]. The best random-effect structure using the restricted maximum likelihood method (REML) was first determined, then followed by the best fixed-effect structure using the maximum likelihood method (ML) [60].

Because the number of detections for each mesocarnivore was below 75 at most sites, we consistently used the type-1 overlap coefficient ∆_1_ to measure the overlap according to suggestions by Meredith and Ridout [56]. The data for road distance and activity were square-root-transformed, and the data for human activity were log-transformed for data normalization to avoid heterogeneity [23]. To avoid random errors, the camera sites with a sampling effort of <60 camera-days were excluded from this analysis.

All analysis was conducted in R (v.4.1.0) [54].

## 3. Results

We gathered the data from September 2016 to December 2020, during which the number of surveyed camera stations progressively increased. We used data from 81, 83, 98, 154, and 187 camera stations from 2016 to 2020, with extensive sampling efforts of 11,060, 15,567, 23,436, 19,271, and 41,729 camera-days each year, respectively. The number of working days of individual stations ranged from 45 to 1384 (mean = 566; sd = 391). On average, the camera stations were located 2137 (±892) m away from human settlements and 1447 (±825) m away from roads. We obtained 120,218 independent detections of 14 wild mammal species, six free-ranging domestic mammals, 36 bird species, and one reptile species (see Tables S1–S3).

**Figure 2:**
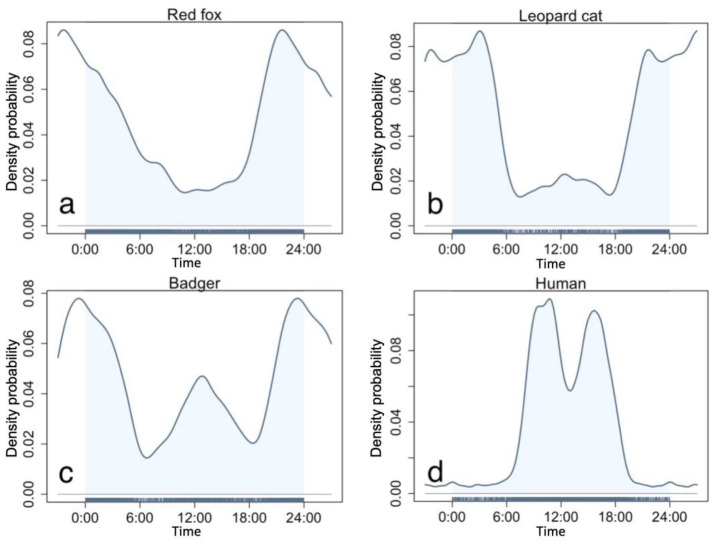
Circadian activity patterns of the three study mesocarnivores and humans. (**a**)—red fox, (**b**)—leopard cat, (**c**)—badger, (**d**)—human.

We obtained 2445 (number of detected stations N = 139), 4893 (N = 147), and 2697 (N = 142) independent detections of leopard cats, red foxes, and badgers, respectively. All three mesocarnivores exhibited mostly nocturnal activity patterns (Figure 2). The red fox was almost strictly nocturnal, being active mainly after 18:00 and before 6:00, with one peak at 20:00 (Figure 2a). The leopard cat was also nocturnal, with two activity peaks at 3:00 and 20:00 (Figure 2b). Its first peak at 3:00 differed to that of the red fox, while the second peak co-occurred. The badgers were cathemeral and demonstrated two activity peaks, one high activity peak around midnight and one relatively lower peak at noon, with little activity at crepuscular hours (Figure 2c). As the three mesocarnivores are mostly nocturnal, their initial activity overlap is considerably high in the study area, with a 0.88 overlap coefficient between leopard cat and red fox, 0.83 between badger and red fox, and 0.84 between leopard cat and badger (see Figure S1a–c). Meanwhile, we recorded 11,306 independent detections of humans from 163 camera stations, with two activity peaks at 8:00–10:00 and 15:00–17:00, respectively (Figure 2d).

### 3.1. Large-Scale Niche Partitioning between Mesocarnivore Pairs

Based on the *K*-means clustering results, human activity was divided into three clusters with 6, 19, and 138 cameras, from high to low human activity (see Table S4 and Figure S2). The high disturbance group has a mean human RAI of 86.3 (sd = 15.6) while the low disturbance group has an RAI of 7.42 (sd = 4.67). Distance to road was divided into four categories with 58, 92, 53, and 11 cameras, ranging from the closest to the farthest (see Table S4 and Figure S3). The closest distance mean is 547.1 m (sd = 237.1) and the farthest is 3580.9 m (sd = 487.1). Village distance was divided into four categories with 35, 66, 65, and 37 cameras, from close to far distances (see Table S4 and Figure S4). The closest distance group has a mean distance of 1005.6 m, and the farthest group has a mean distance of 3531.9 m.

**Figure 3:**
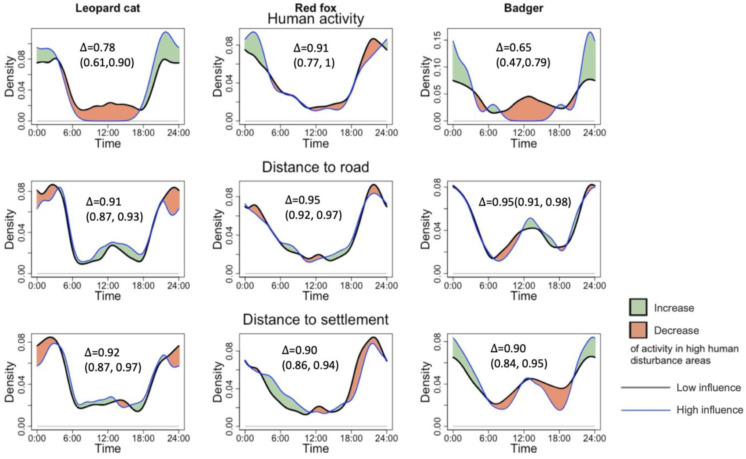
Impacts of human activity, road distance, and village distance on the activity pattern of leopard cats, red foxes, and badgers.

Following our predictions, the leopard cat and badger reduced their diurnal activity at high human activity sites. However, the change was only observable with variable human activity but not human infrastructure. Both species decreased their noon activity significantly with higher human activity and shifted activity to nocturnal hours. The leopard cat ceased to show the noon peak, and instead, displayed one peak at 23:00. The badger significantly decreased daytime activity and exhibited one peak at 24:00, which shifted its activity pattern from cathemeral to nocturnal. In terms of change in overlap coefficient, the badgers showed the most significant decrease in activity overlap with humans by 0.25, while the leopard cat showed a decrease of 0.16. The red fox did not decrease its diurnal activity but shifted its activity peak from 20:00–22:00 at the low human disturbance to 1:00–2:00 at the high disturbance. Road distance and village distance did not affect the activity pattern of the mesocarnivores (see Table 1 and Figure 3).

Because the results indicated that only human activity influenced the temporal patterns of mesocarnivores, only this indicator was analyzed with the degree of overlap between mesocarnivore pairs (Table 1). All the species pairs showed a decrease in the temporal overlap with higher human disturbance, with a decrease of 0.09–0.11. However, none of the species pair overlaps showed a statistically significant difference, as their confidence intervals overlapped (Table 1).

**Figure 4:**
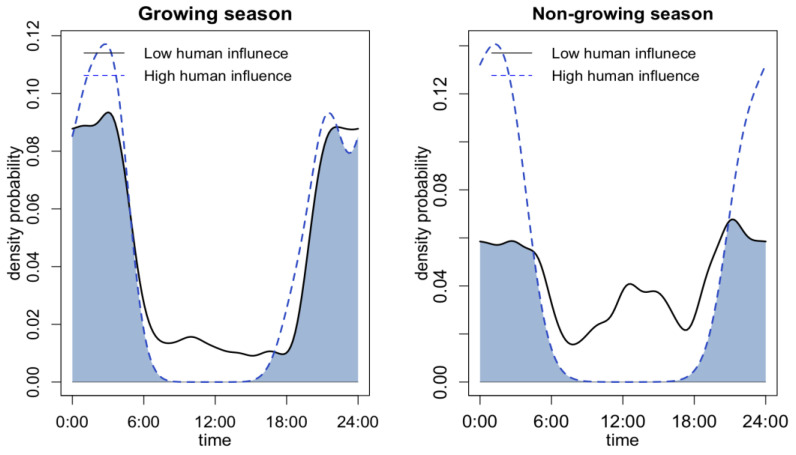
Seasonal differences of leopard cat’s activity pattern under high and low levels of human activity and road distance.

In terms of differences at the seasonal level, only the leopard cat exhibited a clear seasonality as a response to different levels of human activity (Figure 4). Badger activity cannot be compared seasonally due to their winter dormancy. During winter, leopard cats showed a more pronounced diurnal activity peak with low human activity. However, at a high human disturbance level, the leopard cat only displayed nocturnal activity. The overlap between the two activity curves was only 57.21%, indicating a significant shift in activity patterns with different levels of human activity.

### 3.2. Fine-Scale Niche Partitioning between Mesocarnivore Pairs

The results of the linear mixed-effect regression are shown in Table 2. The best model for the three species pairs did not include any of the fixed effect terms, suggesting that at fine spatial scales, human activity and infrastructure are not correlated with the temporal overlap between mesocarnivore species pairs. None of the random and fixed effects can explain the activity overlap at a fine scale between leopard cat and red fox, and red fox and badger. The random intercept of village ID and road ID was selected in the best model of the activity overlap between leopard cat and badger (with an *L* value of 7.53 and a *p*-value of 0.02 when compared with the null model containing only fixed effects using the likelihood ratio test), suggesting that different villages and roads influence the degree of activity overlap and partitioning between the two mesocarnivores.

## 4. Discussion

Our study explores how human disturbances influence temporal activity patterns, thus modifying temporal overlap between mesocarnivores. As expected, the impacts of human pressure on the mesocarnivore avoidance mechanisms are not identical among species [8]. We found that the badgers and the leopard cat shift to nocturnality with increased human activity, in line with our first hypothesis. However, none of the activity overlaps between mesocarnivore pairs demonstrated statistically significant changes under varying human disturbance, contradicting the second hypothesis. The leopard cat displayed significant shifts in activity patterns regarding the growing and non-growing seasons, partially supporting the third hypothesis.

The activity patterns of the three mesocarnivores correspond to previous studies in China [31,34,36,41,61,62]. Following Tsunoda et al.’s categorization [22], all mesocarnivore pairs exhibited high temporal overlap, which is also consistent with previous studies in East Asia [31,36,63]. The two species of badger (i.e., Asian badger and hog badger) were merged in the analysis as they display very similar activity patterns with identical peaks (see Figure S1d). Furthermore, both species inhabit forested environments with similar dietary preferences [64,65], supporting the merge.

In general, our results agree with the global trend toward nocturnality due to human activity [26], with some exceptions [18]. Human infrastructures did not influence the activity patterns of the three mesocarnivores, which corresponds with predictions by Moll et al. [9] and Nickel et al. [66], suggesting that human infrastructure more likely triggers spatial avoidance, while regular human activity impacts temporal responses.

The leopard cats and badgers decreased their diurnal activities when the human activity level was high, likely to avoid negative encounters with humans. Studies in North China suggest that the leopard cat prefers habitats closer to high human-pressure areas [33,67], possibly to avoid the apex predator leopard and the more dominant competitor red fox [68]. Thus, the increased nocturnality may represent a trade-off between avoiding natural competitors using a spatial shield and humans using a temporal shield. In addition, the leopard cat also displays more diurnal activities during the non-growing season at low human-pressure areas, in line with other studies in North China [36]. However, they are exclusively nocturnal in high human-pressure areas, possibly to avoid humans despite the higher need for diurnal prey during winter [69].

On the contrary, the badgers tend to spatially avoid humans, for instance avoiding dogs [42], avoiding settlements and roads [70], and building setts away from human pressure [71,72]. Therefore, the shift to nocturnality may represent the badgers’ sensitive responses to human disturbance. However, more research would be needed to verify the mechanisms for the activity shifts.

The red fox did not change its activity pattern with varied human disturbances, corresponding with other studies of human disturbances [9,62]. This suggests either that it is more tolerant to temporal human disturbances, or that its initial strong nocturnal preference leaves little space for further shifts. Most research in East Asia found the red fox’s diet to be primarily constituted of rodents [35,72,73,74], partially explaining the red fox’s nocturnal preferences under little human disturbances.

Temporal overlap between mesocarnivores did not respond to human disturbance, both on a large and on a fine scale. As Sévêque et al. pointed out in a meta-analysis [8], changes to temporal overlap by human disturbances are not unidirectional across ecosystems. Given the high initial overlap between the three mesocarnivores (75% to 89%) at both the low and high human disturbance area, the mesocarnivores may have established a long-run mechanism regardless of location-specific human pressure due to decades of human activities around the reserve and surrounding areas.

Although the changes in the overlap coefficient we observed were not statistically significant, the animals’ activity peaks may have shifted to accommodate the increased nocturnality. For instance, at the sites with a high level of human disturbance, while the leopard cat displays an activity peak at 20:00–22:00, the red fox shifted its activity peak from 20:00–22:00, which overlapped with the peak of the leopard cat, to 1:00–2:00 (see Figure 3). As a result, temporal overlap remained at a similar level with non-overlapping activity peaks at each human activity level.

Furthermore, the lack of changes in activity overlap may be mitigated by other niche dimensions. For instance, Zhang et al. found that sympatric mesocarnivores coexist by partitioning space and time in different seasons, to achieve coexistence while having high overlap in certain dimensions in different seasons [36]. Reduced occurrence of competitors in high human disturbance areas may also reduce competition and the need for temporal segregation [8,21]. The mesocarnivores may also reactively respond to the presence of other mesocarnivores [8,21], thereby avoiding encounters and direct competition. Across East Asia, the leopard cat and red fox have a diverse and wide diet (red fox: [75,76,77]; leopard cat: [70,74,78]). For instance, red fox in areas with a high human footprint Index consumes more fruits and birds [76]. This spatial and trophic plasticity may also facilitate coexistence under human pressure.

Despite the lack of changes in temporal overlap, diel activity shifts and reduced diurnal activities can still create substantial fitness costs by altering resource use and patterns of competition [26]. The three mesocarnivores share similar dietary and spatial preferences in East Asia (trophic: e.g., [35,74]; spatial: e.g., [31,34,79]), fulfilling requirements of the competitive exclusion principle [80]. Therefore, intensified nocturnality, while not increasing temporal overlap, may add pressure to the other two niche dimensions. By restricting circadian rhythms, human activity may narrow trophic niche breadth and enhance dietary niche overlap [3]. The badgers and leopard cats rely heavily on both nocturnal (e.g., rodents and insects) and diurnal (e.g., birds) prey [74], as well as large quantities of plants usually consumed during the day [64,65]. Restriction to diurnal activities may reduce fitness [19] by degrading their ability to hunt varied prey types, thus changing the biotic composition of lower trophic levels. This would particularly affect the leopard cats during winter, as resources are scarcer [67] and ecologically similar species usually choose to segregate the consumption of scarce resources [22,81]. However, under high human disturbance, leopard cats display more nocturnal behaviors, suggesting a compromise in hunting strategies and potential deterioration of the sympatry mechanism in a resource-scarce season.

The shift to nocturnality may also decrease the fitness of other species. Although the masked palm civet is not examined in our study due to low detection rates, they may compete with the badgers, as they are found to display spatial avoidance [31] with close dietary preferences [82,83], seasonal activity [36], and diel activity patterns [31]. As the more dominant and abundant species, the badgers’ shift to nocturnality increases temporal overlap with the civet, which potentially decreases the civet’s fitness as the more subordinate predator.

There are several limits in our study that should be considered in further research in this area and elsewhere on mesocarnivores. In the TQS reserve and the surrounding areas, besides the anthropogenic factors we considered in this study, there are various types of human disturbance, such as heavily cultivated lands, historically clear-cut areas, and green energy infrastructures (e.g., hydropower and solar energy) that may deteriorate habitats [84]. Future studies may consider generating an integrated index, similar to the human footprint index [85], to measure human disturbance at fine resolution. Furthermore, camera-trapping can be subjected to detecting biases [86] associated with camera orientation, season, time, and animal traits such as body size and behavioral characteristics [87,88], leading to varied detectability across different sites [89]. For instance, mesocarnivores may scurry faster at sites with high human pressure, resulting in lower detectability (e.g., [90]. An analytical approach that can deal with the varied detection probability, such as occupancy modeling, may be adopted to overcome this issue. The smoothed kernel density function with the overlap tool may also introduce biases in the smoothing process [86], as a different selection of *K*-max values may alter the smoothing results and the overlap coefficient. Cross-studies involving satellite-tracking collars may improve our understanding of the mechanism driving fine-scale spatiotemporal partitioning between mesocarnivores.

## 5. Conclusions

Activity pattern is a focal dimension of an animal’s ecological niche [14]. Understanding the temporal interactions among mesocarnivores predicts species vulnerable to human disturbance and weighs risks to wildlife communities, and informs conservation and management decisions that preserve the dynamics of a carnivore community in a human-dominated landscape [16,91]. Our study provides strong evidence that some species of wildlife shift to nocturnality under high human disturbance and suggests that protected areas at the wildlife–urban interface may not always maintain ecological processes [3]. When making conservation decisions, it is important to consider the impacts of human activity as well as the infrastructure on the landscape and incorporate considerations of human influences on temporal niche partitioning. In the future, more studies should be focused on the interplay of the three niche dimensions and their subsequent effects at the individual, population, and community levels to inform more effective conservation strategies.

## Figures and Tables

**Table 1:** Degree of overlap between species pair and overlap with humans at two human activity levels, measured using type-4 overlap coefficient (∆_4_). The *p*-value of the difference between the two distributions was determined using Welch’s T-test, and coefficients showing significance between activity levels are in bold.

Species Pair	$\mathrm{Low}\;\mathrm{Human}\;\mathrm{Activity}\;\Delta_{4}\;(95\%\;\mathrm{CI})$	$\mathrm{High}\;\mathrm{Human}\;\mathrm{Activity}\;\Delta_{4}\;(95\%\;\mathrm{Cl})$	Difference	*p*-Value
**Badger–human**	**0.41 (0.39, 0.43)**	**0.16 (0.05, 0.31)**	**−0.25**	**0.000**
**Leopard cat–human**	**0.27 (0.25, 0.29)**	**0.11 (0.03, 0.21)**	**−0.16**	**0.000**
Red fox–human	0.27 (0.25, 0.29)	0.27(0.13, 0.43)	0.00	0.730
Leopard cat–badger	0.86 (0.83, 0.88)	0.75(0.50, 0.94)	−0.11	0.324
Red fox–leopard cat	0.89 (0.87, 0.91)	0.80(0.59, 0.97)	−0.09	0.365
Red fox–badger	0.84 (0.82, 0.86)	0.76(0.53, 0.96)	−0.08	0.471

**Table 2:** Mixed-effect regression model describing the influence of covariates and random effects on temporal activity overlap between leopard cats, red foxes, and badgers.

	Species Pairs					
	Leopard Cat, Red Fox		Leopard Cat, Badger		Red Fox, Badger	
**Random Effects** Village ID intercept Road ID intercept	$\sigma^{2}\;\left(\mathrm{SD}\right)$		$\sigma^{2}\;\left(\mathrm{SD}\right)$		$\sigma^{2}\;\left(\mathrm{SD}\right)$	
			0.000 (0.000)			
			0.003 (0.055)			
Residual **Fixed Effects**			0.033 (0.182)			
	β (SE)	*t* value	β (SE)	*t* value	(SE)	*t* value
(Intercept) Road distance Village distance Human activity	0.606 (0.016)	36.946	0.609 (0.018)	33.773	0.606 (0.016)	36.946
					
					
					

## Data Availability

The camera-trapping data are owned by the Chinese Felid Conservation Alliance and Beijing Normal University, and are authorized for use in this study.

## Supplementary Materials

The following supporting information can be downloaded at: https://www.mdpi.com/article/10.3390/ani13040688/s1, Table S1: List of mammal species detected during the camera-trapping survey in the study area, Shanxi province, from 2016 to 2020; Table S2: List of bird species detected during the camera-trapping survey in the study area, Shanxi province, from 2016 to 2020; Table S3: List of reptile species detected during the camera-trapping survey in the study area, Shanxi province, from 2016 to 2020; Table S4: The *K*-means grouping results for human activity, distance to road, and distance to village variables, where bolded groups are selected for analysis; Figure S1: The circadian activity overlaps between mesocarnivore pairs. a, b, and c—the activity overlap between leopard cat, red fox, and badger, d—the activity pattern between the two badger species, the hog badger, and the Asian badger; Figure S2: The seven analysis methods used to select the optimal *K*-means grouping for human activity; Figure S3: The seven analysis methods used to select the optimal *K*-means grouping for distance to road; Figure S4: The seven analysis methods used to select the optimal *K*-means grouping for distance to village.
