# Supplementary material for: Temporal Response of Mesocarnivores to Human Activity and Infrastructure in Taihang Mountains, Central North China: Shifts in Activity Patterns and Their Overlap
Source: Animals (Basel). 2023 Feb 16;13(4):688. doi: 10.3390/ani13040688 (PMC9952777; doi:10.3390/ani13040688)
Supplement: Supplementary file 1 [file animals-13-00688-s001.zip › animals-2074357-supplementary.pdf]

SUPPLEMENTARY MATERIALS

Table S1. List of mammal species detected during the camera-trapping survey in the study area, Shanxi province, from 2016-2020.

Taxa	Species	Scientific name [a]	Distribution type [b]	IUCN Red List category [c]	No. of detection	RAI [d]
EULIPOTYPHLA						
Erinaceidae	Amur hedgehog	*Erinaceus amurensis*	P	LC	19	0.02
RODENTIA						
Sciuridae	Eurasian red squirrel	*Sciurus vulgaris*	P	LC	15	0.02
	Pere David's rock squirrel	*Sciurotamias davidianus*	P	LC	14,515	13.98
	Siberian chipmunk	*Tamias sibiricus*	P	LC	718	0.69
LAGOMORPHA						
Leporidae	Tolai hare	*Lepus tolai*	P	LC	9,216	8.88
CARNIVORA						
Canidae	Red fox	*Vulpes vulpes*	P	LC	4,893	4.71
	Domestic dog	***Canis lupus familiaris***	**-**	**-**	**1,639**	**1.58**
Mustelidae	Siberian weasel	*Mustela sibirica*	P	LC	87	0.08
	Asian badger	*Meles leucurus*	P	LC	234	0.23
	Hog badger	*Arctonyx collaris*	O	NT	291	0.28
	Unidentified badger		-	-	2,174	2.09
Viverridae	Masked palm civet	*Paguma larvata*	O	LC	42	0.04
Felidae	Leopard	*Panthera pardus*	P	VU	2,189	2.11
	Leopard cat	*Prionailurus bengalensis*	O	LC	2,445	2.35
	Domestic cat	***Felis catus***	**-**	**-**	**51**	**0.05**
ARTIODACTYLA						
Suidae	Wild boar	*Sus scrofa*	P	LC	5,478	5.28
Cervidae	Siberian roe deer	*Capreolus pygargus*	P	LC	9,221	8.88
Bovidae	Cattle	***Bos taurus***	**-**	**-**	**35,351**	**34.05**
	Goat/Sheep	***Capra aegagrus hircus/Ovis aries***	**-**	**-**	**174**	**0.17**
PERISSODACTYLA						
Equidae	Horse	***Equus caballus***	**-**	**-**	**2**	**0.00**
	Donkey	***Equus africanus asinus***	**-**	**-**	**135**	**0.13**

[a] Bolded species represent domestic mammals.
[b] P: Palaearctic species; O: Oriental species.
[c] LC: least concern; NT: near threatened; VU: vulnerable, NE: not evaluated.
[d] RAI: calculated as the number of independent detections per 100 camera-days.

Table S2. List of bird species detected during the camera-trapping survey in the study area, Shanxi province, from 2016-2020.

Taxa	Species	Scientific name [a]	Distribution type [b]	IUCN Red List category [c]	No. of detection	RAI [d]
GALLIFORMES						
Phasianidae	Ring-necked pheasant	*Phasianus colchichus*	P	LC	14,049	13.53
	Koklass pheasant	*Pucrasia macrolopha*	P	LC	30	0.03
	Brown-eared pheasant	*Crossoptilon mantchuricum*	P	VU	1	0.00
	Golden pheasant	*Chrysolophus pictus*	P	LC	2	0.00
PICIFORMES						
Picidae	Great spotted woodpecker	*Dendrocopos major*	P	LC	10	0.01
	Grey-headed woodpecker	*Picus canus*	P	LC	14	0.01
STRIGIFORMES						
Strigidae	Long-eared owl	*Asio otus*	P	LC	2	0.00
COLUMBIFORMES						
Columbidae	Oriental turtledove	*Streptopelia orientalis*	P	LC	27	0.03
	Spotted dove	*Streptopelia chinensis*	P	LC	1	0.00
CHARADRIIFORMES						
Scolopacidae	Eurasian woodcock	*Scolopax rusticola*	P	LC	1	0.00
CICONIIFORMES						
Accipitridae	Eurasian sparrowhawk	*Accipiter nisus*	P/O	LC	3	0.00
	Northern goshawk	*Accipiter gentilis*	P/O	LC	1	0.00
PASSERIFORMES						
Corvidae	Eurasian jay	*Garrulus glandarius*	P	LC	71	0.07
	Spotted nutcracker	*Nucifraga caryocatactes*	P	LC	241	0.23
	Red-billed blue magpie	*Urocissa erythroryncha*	O	LC	626	0.60
	Oriental magpie	*Pica serica*	P	LC	58	0.06
Turdidae	Scaly thrush	*Zoothera dauma*	P	LC	58	0.06
	Naumann's thrush	*Turdus naumanni*	P	LC	1	0.00
	Chinese thrush	*Turdus mupinensis*	P	LC	223	0.21
	Eyebrowed thrush	*Turdus obscurus*	P	LC	1	0.00
	Blue whistling thrush	*Myophonus caeruleus*	O	LC	17	0.02
	Daurian redstart	*Phoenicurus auroreus*	P	LC	10	0.01
	Red-flanked bluetail	*Tasiger cyanurus*	P	LC	9	0.01
Timaliidae	Plain laughingthrush	*Garrulax davidi*	P	LC	12	0.01
Sylviidae	Oriental reed warbler	*Acrocephalus orientalis*	P	LC	1	0.00
Paridae	Great tit	*Parus major*	P/O	LC	6	0.01

	Japanese tit	*Parus minor*	P/O	NE	4	0.00
	Marsh tit	*Parus palustris*	P	LC	1	0.00
	Yellow-bellied tit	*Parus venustulus*	P	LC	1	0.00
Fringilidae	Brambling	*Fringilla montifringilla*	P	LC	19	0.02
	Siberian long-tailed rose-finch	*Carpodacus sibiricus*	P	LC	11	0.01
Emberizida	Yellow-throated bunting	*Emberizida elegans*	P	LC	1	0.00

Table S3. List of reptile species detected during the camera-trapping survey in the study area, Shanxi province, from 2016-2020.

**Taxa**	**Species**	**Scientific name** [a]	**Distribution type** [b]	**IUCN Red List category** [c]	**No. of detection**	**RAI** [d]
SQUAMATA						
Viperidae	North China pitviper	*Gloydius stejnegeri*	P	NE	1	0.00

Table S4. The K-means grouping results for human activity, distance to road, and distance to village variables, where bolded groups are selected for analysis.

Variable	Number of groups	Group size	Group mean (Standard deviation)
Human activity	**1**	**7**	**7.46 (4.67)**
	2	19	37.07 (8.35)
	**3**	**137**	**90.22 (17.69)**
Distance to road (m)	**1**	**58**	**547.13 (237.06)**
	2	92	1331.13 (212.86)
	3	63	2305.77 (298.43)
	**4**	**11**	**3580.85 (487.14)**
Distance to village (m)	**1**	**46**	**1005.64 (262.59)**
	2	65	1745.76 (225.71)
	3	66	2635.93 (241.71)
	**4**	**37**	**3531.88 (377.69)**

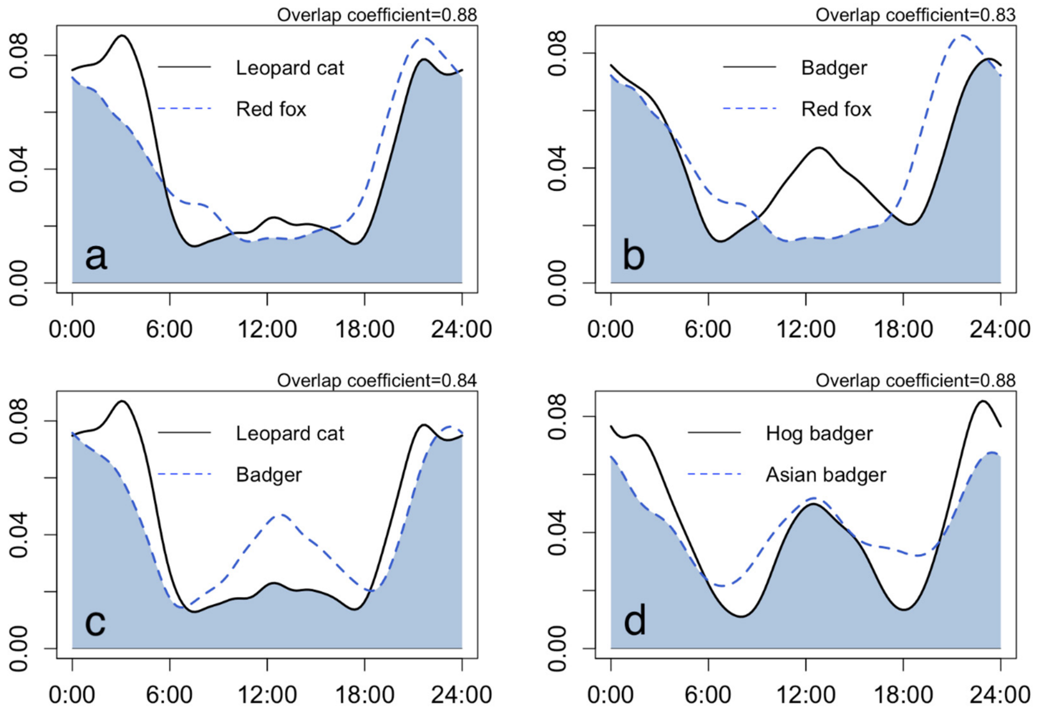

Figure S1. The circadian activity overlaps between mesocarnivore pairs. a, b, and c - the activity overlap between leopard cat, red fox, and badger, d- the activity pattern between the two badger species, the hog badger and Asian badger.

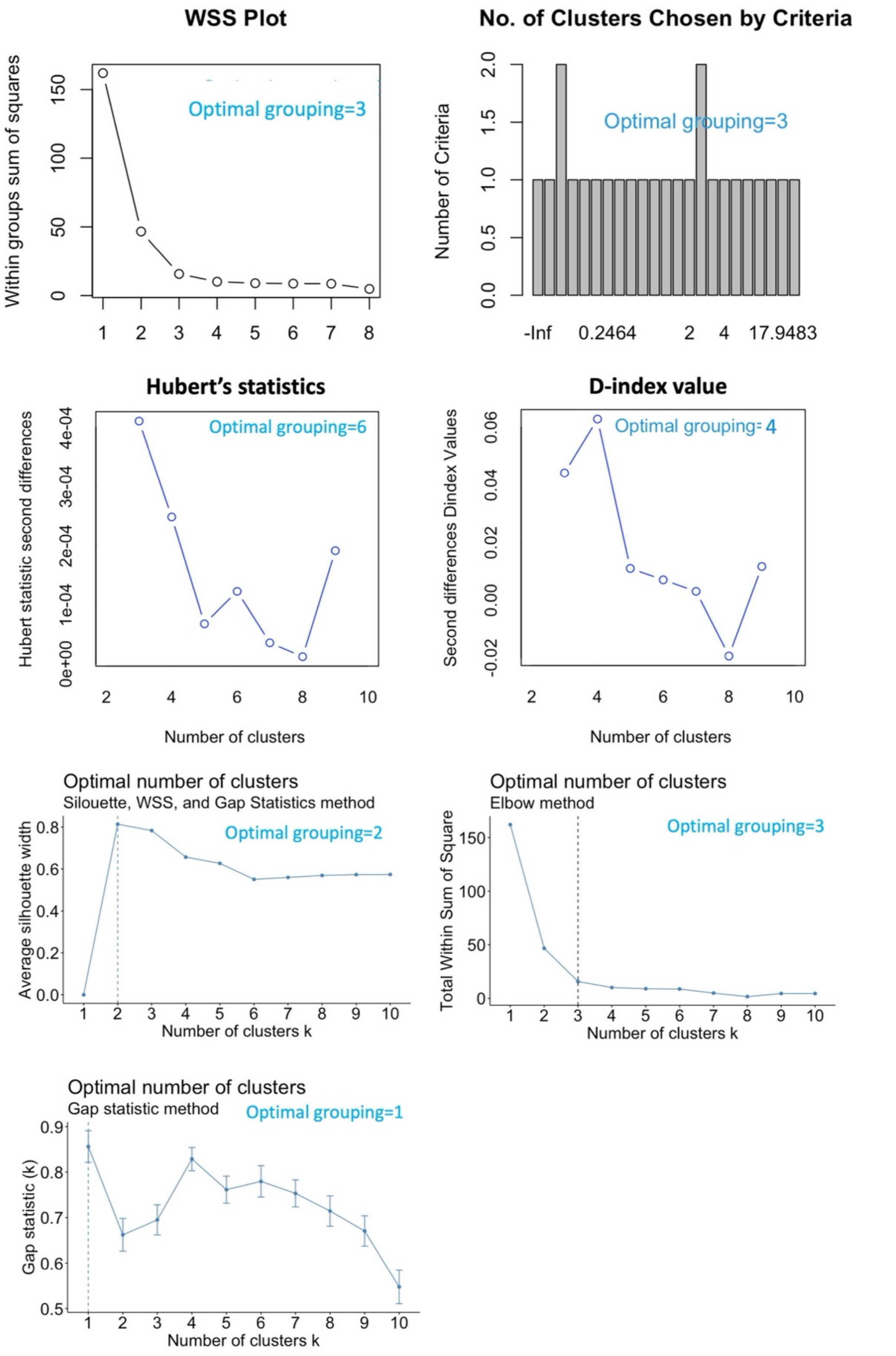

Figure S2. The seven analysis methods used to select the optimal *K*-means grouping for human activity.

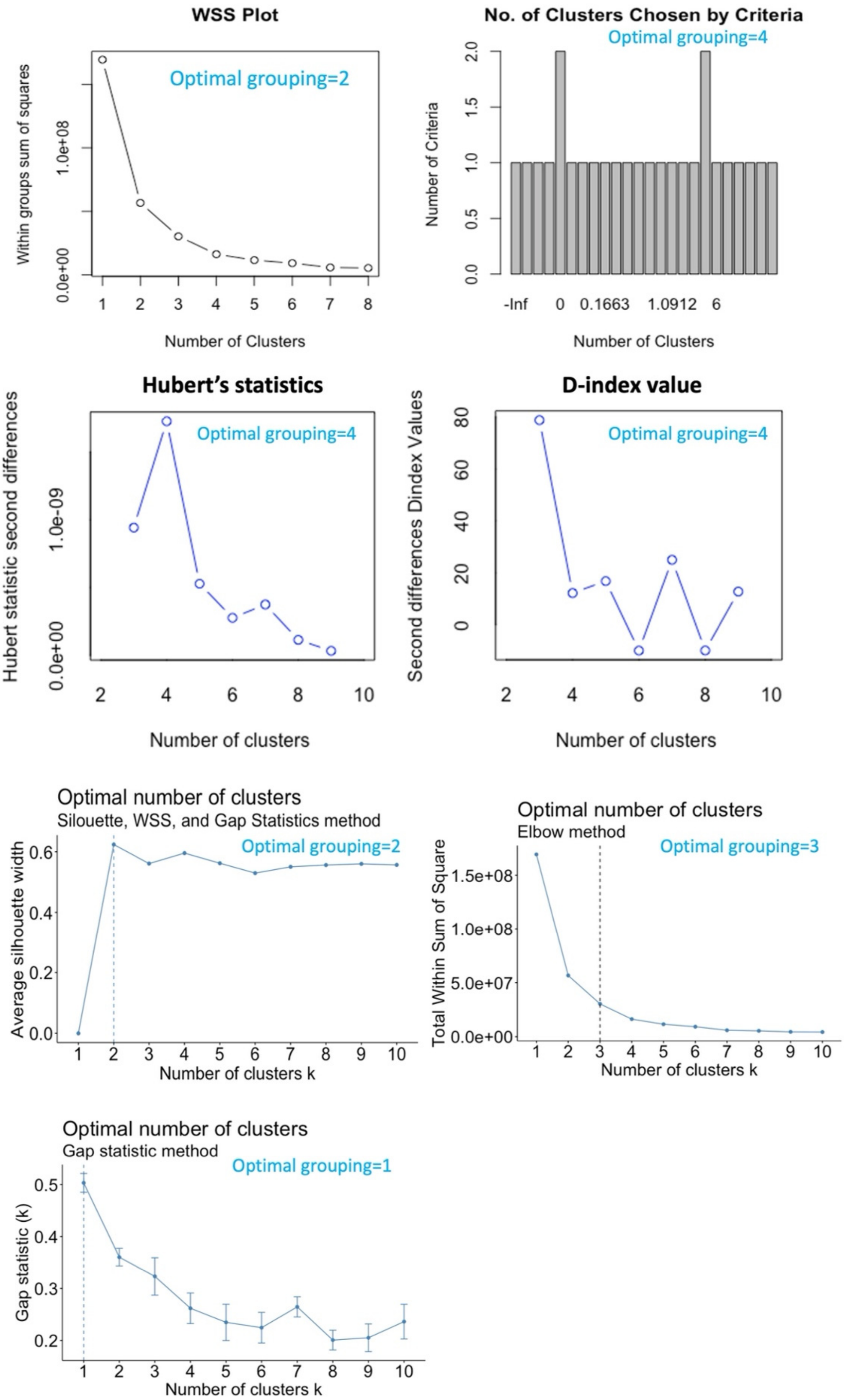

Figure S3. The seven analysis methods used to select the optimal *K*-means grouping for distance to road.

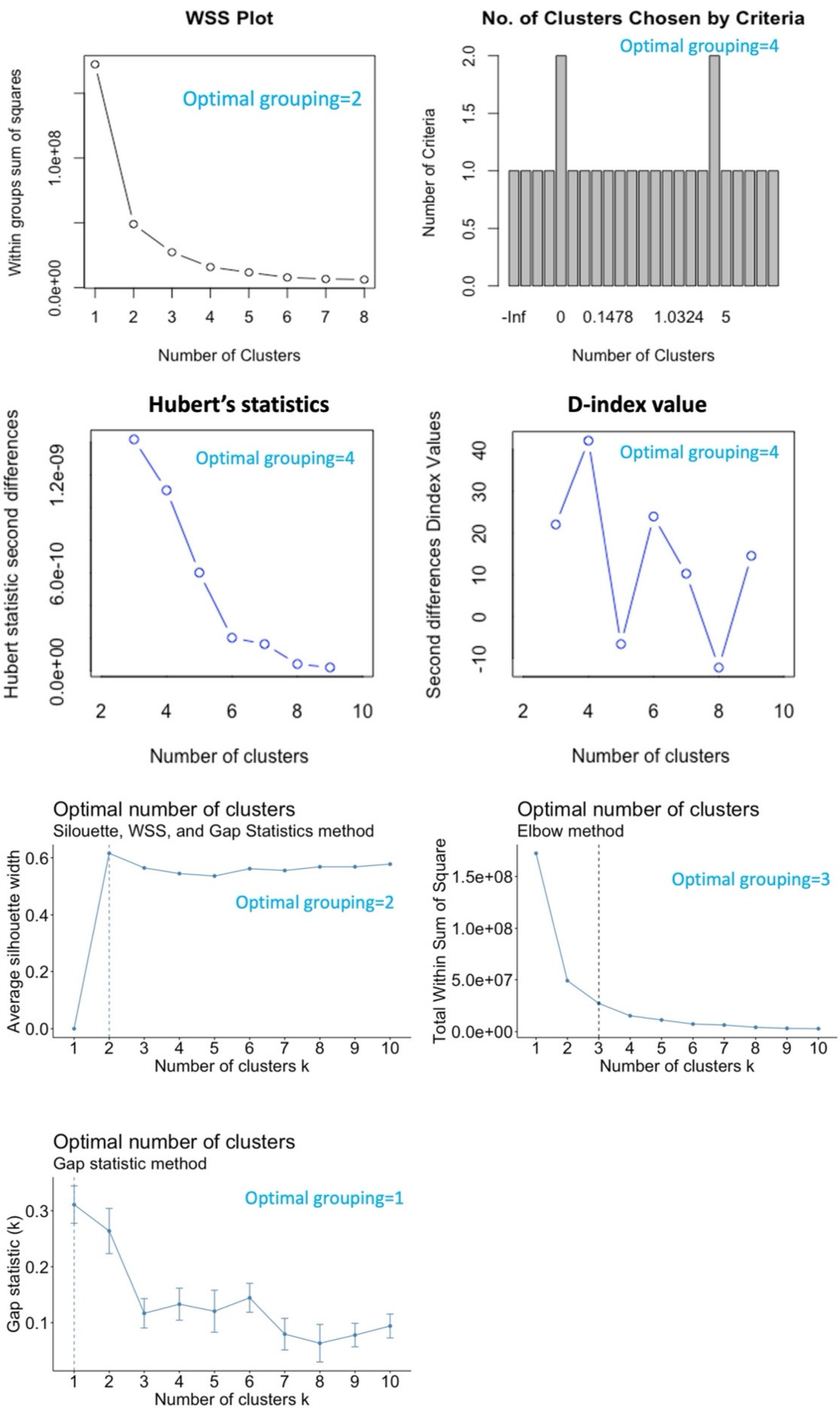

Figure S4. The seven analysis methods used to select the optimal *K*-means grouping for distance to village.
